# Highly Sensitive, Engineered Magnetic Nanosensors to Investigate the Ambiguous Activity of Zika Virus and Binding Receptors

**DOI:** 10.1038/s41598-017-07620-y

**Published:** 2017-08-07

**Authors:** Tyler Shelby, Tuhina Banerjee, Irene Zegar, Santimukul Santra

**Affiliations:** Department of Chemistry, Pittsburg State University, 1701 S. Broadway Street, Pittsburg, KS 66762 USA

## Abstract

The aim of this research is twofold: 1) to shed light on zika’s binding and entry mechanism while 2) demonstrating the effectiveness of our magnetic relaxation platform to achieve this goal. Magnetic relaxation-sensitive nanoparticles (MRNPs) are used in a novel fashion to analyze binding interactions between the zika envelope protein (ZENV) and proposed host cell receptors: AXL, HSP70, and TIM-1. Computational analysis is also utilized to examine these binding interactions for the first time. In addition, the role of crizotinib as a potential binding inhibitor is demonstrated and the possibility of ligand-independent phosphatidylserine-mediated binding is explored. Our findings suggest that while the extracellular domain of AXL has the highest affinity for ZENV; HSP70, TIM-1, and phosphatidylserine might also play active roles in zika tropism, which offers a potential explanation for the variety of zika-associated symptoms. This is, to our knowledge, the first time that MRNPs have been used to examine and quantify host-zika interactions. Our magnetic relaxation platform allows for timely and sensitive analysis of these intricate binding relationships, and it is easily customizable for further examination of additional host-pathogen interactions.

## Introduction

Zika has recently become the center of much media attention, as well as the focus of a number of research studies working towards a deeper understanding of viral structure and pathogenesis^1^, and ultimately, more efficient diagnostic and treatment techniques^2^. One of the most vital mechanisms for the propagation of zika virus (ZIKV) is the binding and entry stage. While there is still much to be determined about this key stage, it has been demonstrated that the initial interactions between ZIKV and receptors expressed in various host cell populations is a critical determinant of ZIKV tropism^3^. It is for this reason that we have begun to investigate these host-pathogen interactions using our ultra-sensitive magnetic relaxation nanoplatform^4, 5^.

Often, infection by ZIKV results in mild to non-observable symptoms^6^. However, what makes zika an outlier when compared to other viruses is the rare appearance of additional symptoms, including microcephaly and Guillain-Barre syndrome^6, 7^. This unexplained array of symptoms may be the result of promiscuous and ambiguous activity of ZENV. Recent articles have proposed that the preferred host cell receptor is AXL^3, 8–13^, a receptor commonly associated with other flaviviruses^8^. AXL is heavily expressed in radial glia stem cells found in the fetal cerebral cortex, which is an area associated with microcephaly^8^. In addition, AXL has been shown to have both ligand dependent and ligand-independent activation behavior^14–16^. Ligand independent activation of AXL is known to occur with its overexpression and does not depend upon its kinase activity^15^. Based upon these reports, AXL was selected for this study. HSP70 was also selected for testing based upon its ability to facilitate binding of the dengue virus^17, 18^. The genetic similarity between zika and dengue and the structural similarity between their envelope proteins suggest that HSP70 may also play a role in ZIKV propagation. Furthermore, HSP70 has been linked to the neurotropism of Japanese encephalitis virus^19^, inferring that it may also play a role in the neurotropism of ZIKV. Lastly, it has been indicated that ZIKV may also interact with TIM-1^8–10^, the receptor known for its roles in Ebola and Dengue viral binding^17, 20, 21^, and it was selected for these studies as well.

In addition to studying the interactions between these receptors and ZENV, we wished to examine parameters that affect the binding process, such as the presence of crizotinib (Cz) or phosphatidylserine (PS), as well as the effects of temperature^22^ and pH^23^. Cz is an ATP mimic that has been shown to interfere with AXL kinase activity^24^. PS has been associated with apoptotic mimicry and host-cell entry in a number of viruses, including other flaviviruses^25–27^, and may therefore have a role in ZIKV binding and entry. Finally, in contrast to other flaviviruses, ZIKV remains infective at a wider range of temperatures^22^ and pH values^23^, which have been further characterized in this work.

## Results and Discussion

### Receptor Specificity

**Figure 1:**
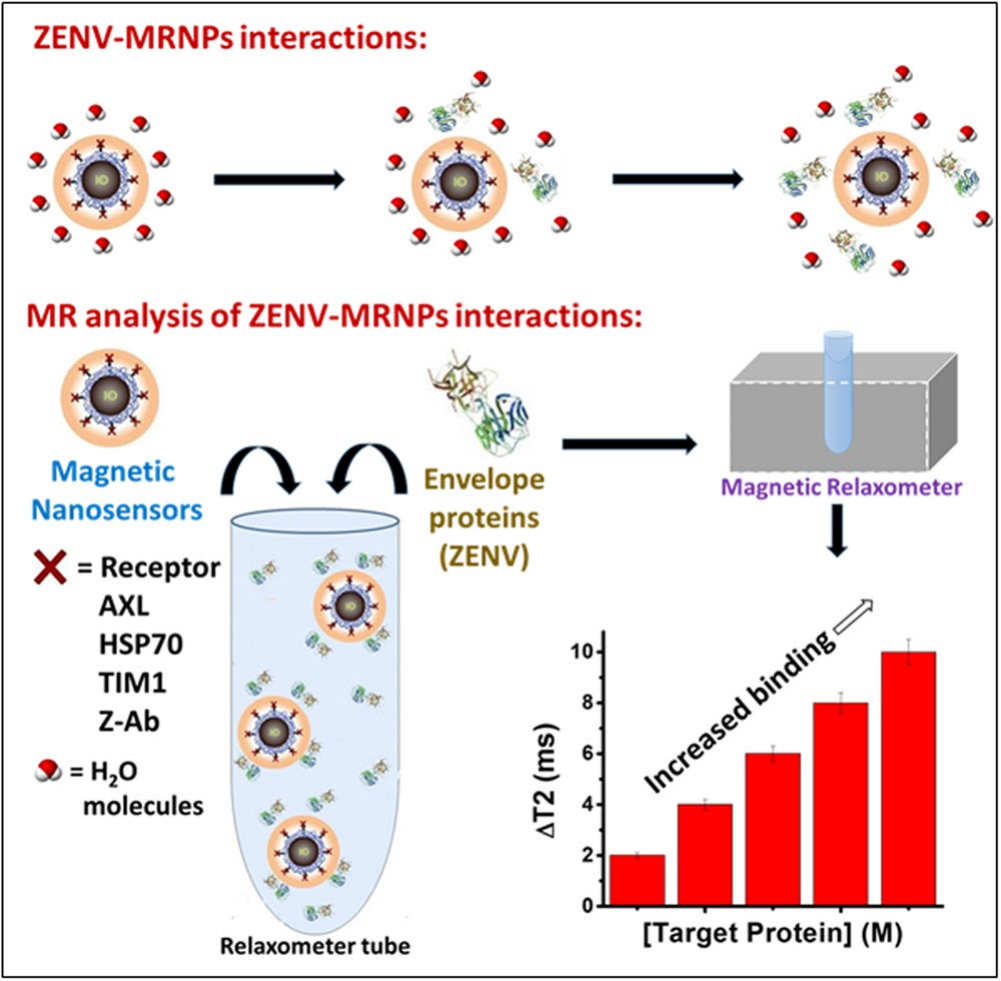
ZENV-MRNPs interactions: Schematic representation of neighboring water proton displacement by ZENV-MRNPs interactions in solution. MR analysis of ZENV-MRNPs interactions: Magnetic relaxation (MR) nanosensor for the assessment of these interactions and receptor activities.

Using our magnetic relaxation platform featured in Fig. 1, we first examined the binding between ZENV and the selected receptors. Customization of iron oxide nanoparticles^28^ (IONPs) via protein-conjugation allows for the sensitive detection of binding between selected proteins. Receptors and antibodies are conjugated to the surface carboxylic acid groups of IONPs using EDC/NHS chemistry which functionalizes the nanoparticles (MRNPs) for targeted binding with ZENV. When binding occurs between the MRNPs and ZENV in solution, the surrounding water molecules are displaced from the nanoparticle and magnetic relaxation times (T2) increase (raw data shown in supplemental information, SI, Figure S1). As the magnetic core of the nanoparticle is separated from surrounding water protons, its effect on the nuclei spin of each water proton is lessened, causing the increase in T2 values (Fig. 1). For the sake of simpler representation, T2 data is normalized to magnetic relaxation (∆MR) values, according to the equation:

$${\rm{\Delta }}MR=\frac{{\rm{\Delta }}T2}{{\rm{\Delta }}T{2}_{Max}},$$

previously reported^5^ and further explained in the SI. This binding is verified by the addition of “free” receptors (receptors that are not bound to IONPs), ensuring that the binding is due to specific interactions between ZENV and the selected target proteins, and not the nanoparticle itself.^5^ Competition values are also normalized according to:

$${\rm{\Delta }}M{R}_{Comp}=\frac{{\rm{\Delta }}T{{2}_{Max}}^{0}-{\rm{\Delta }}T{2}_{Comp}}{{\rm{\Delta }}T{{2}_{Max}}^{0}},$$

which is further explained in the supporting information.

**Figure 2:**
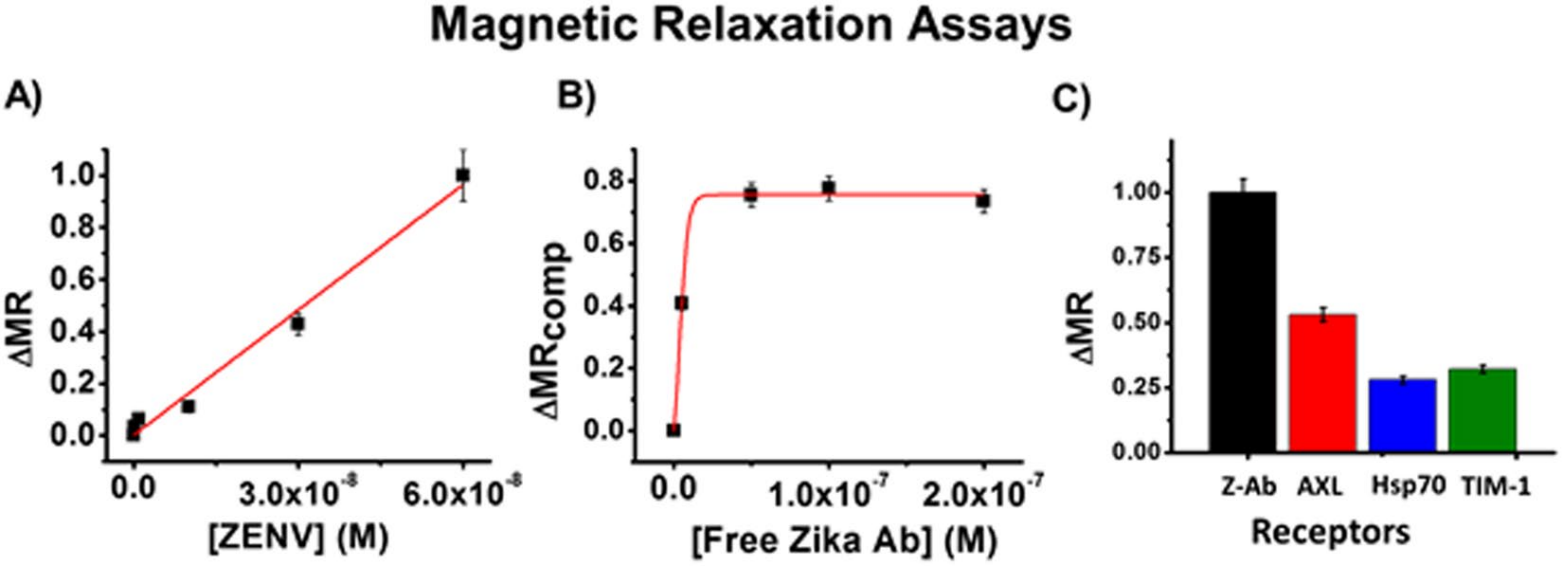
(**A**) Z-Ab-MRNPs (1.5 mM) were incubated with ZENV (0 to 6E^−8^ M) for 30 minutes at 25 °C, resulting in increasing ∆MR values as binding increased between Z-Ab-MRNPs and ZENV. (**B**) Z-Ab-MRNPs were incubated with ZENV (3E^−8^ M) and free Z-Ab (0 to 2E^−7^ M). (**C**) MRNPs conjugated with Z-Ab, AXL, HSP70, and TIM-1 were incubated with ZENV (3E^−8^ M). As shown, AXL exhibited the strongest binding among the proposed receptors. Average values of three measurements are depicted ± standard error.

Before we began to examine the interactions between ZENV and our selected receptors, we wished to demonstrate the capabilities of our platform to sensitively analyze minute molecular interactions using a known protein-protein relationship: antibody and antigen. For this purpose, we functionalized MRNPs with an anti-zika IgG monoclonal antibody (Z-Ab) and incubated them in PBS solutions (1X, pH = 7.4) with increasing concentrations of ZENV (0 to 6E^−8^ M) for 30 minutes at 25 °C. This data is represented in Fig. 2A. As shown, there was a strong correlation between ∆MR values and ZENV concentration, indicative of binding. A negative control test was conducted using hemagglutinin instead of ZENV, and is shown in the SI, Figure S2. A competition assay was then conducted by incubating Z-Ab-MRNPs (1.5 mM) in PBS (1X, pH = 7.4) with ZENV (3E^−8^ M) and free Z-Ab (0 to 2E^−7^ M) for 30 minutes at 25 °C. This data (Fig. 2B) verifies that the interaction between Z-Ab-MRNPs and ZENV is due to specific interactions between Z-Ab and ZENV. Additionally, by determining the point at which 50% of ZENV molecules in solution were bound by Z-Ab, we were able to determine an apparent K_d_, as previously reported^5^. This apparent K_d_ was ~1.1E^−8^ M.

After we verified the effectiveness of our platform using the antibody, we began to examine the interactions between ZENV and our selected receptors. MRNPs were functionalized with the extracellular domain of AXL, HSP70, and TIM-1, as previously described^4, 5^. Our Z-Ab-MRNPs were used in order to comparatively analyze the binding between the suspected receptors. Binding assays were conducted by incubating receptor-bound MRNPs with ZENV (3E^−8^ M) in PBS (1X, pH = 7.4) for 30 minutes at 25 °C and recording T2 values after this time. These T2 values were compared to those collected from baseline solutions that contained only MRNPs in PBS. The final comparable ∆MR values are presented in Fig. 2C. Among the selected receptors, AXL exhibited the strongest binding, which is in accordance with what has been presented in previous literature. However, it is important to note that there is a significant degree of binding between ZENV and both HSP70 and TIM-1, supporting our hypothesis that ZIKV is able to interact with various receptors. Duplicate assays were conducted in human plasma solutions, and these similar results are reported in the SI, Figure S3. Simple competition assays were conducted to ensure that the binding between these MRNPs and ZENV was due to receptor specificity, and these results are shown in SI Figure S4.

### Computational Analysis of AXL-ZENV interaction

**Figure 3:**
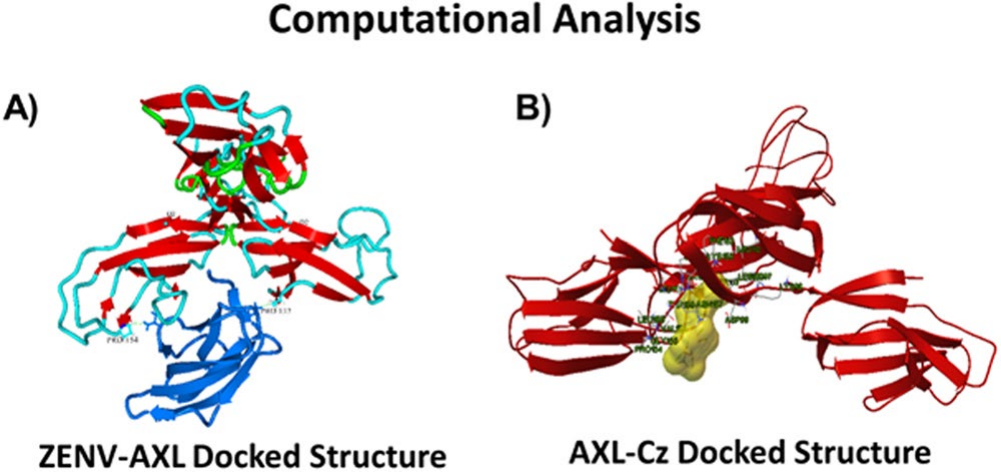
(**A**) ZENV-DIII-AXL-D1D2 docked structure. The ZENV-DIII domain is shown in blue. ZENV-DIII lies in a pocket of AXL-D2 residues, resulting in a binding equilibrium value for ZENV-AXL of 2.8E^−6^ M. (**B**) Vina-generated docking structure of Crizotinib with AXL. The corresponding binding free energy = −7.3 kcal/mol when Crizotinib binds to the same pocket as ZENV-DIII.

After collecting these primary binding data, we began to further examine the binding relationship between AXL and ZENV. First, we used computational docking analysis to further examine and quantify the interaction between ZENV and AXL. We also pursued computational analysis in order to screen for molecules capable of inhibiting this interaction. The structure and coordinates for ZENV (SI, Figure S5) were obtained from the coordinate file for ZENV-DIII bound to an antibody (PDB ID: 5KVE). The structure for domains I-II of AXL were created by using BLAST search to screen the PDB structural database (http://www.rcsb.org) for structures sharing primary sequence identity with AXL-Ig-D1D2. The results indicated that Tyro3 tyrosine kinase (PDB ID: IRHF) shared 71% primary sequence identity. The IRHG crystal structure was subsequently used as a template to construct the 3D structure of the AXL-D1D2 by comparative modeling followed by loop refinement using Modeller 9.17^29^. The generated structure was further energy minimized using the “RepairPDB” option of the FoldX algorithm plugin in Yasara View Version 16.9.23^30^ which is used to repair torsion angle distortions and Van der Waals clashes. The final structure is shown in SI Figure S6. Molecular docking of ZENV-DIII to AXL-D1D2 was conducted by first using GRAMM-X Protein-Protein Docking Web Server v.1.2.0 to obtain a docking complex with a reasonable guess of the starting position^31^. The resulting complex with the most stable energy was then submitted to the Rosetta online server (http://rosie.rosettacommons.org)^32^. The docking was performed using the protein-protein Docking 2 protocol which determines the structures of protein-protein complexes by using rigid body perturbations. Two hydrophobic interactions between His152 on AXL-D1D2 and Val 358 of ZENV-DIII and between Pro137 on AXL-D1D2 and Asp333 of ZENV-DIII are indicated in Fig. 3A.

We then used further computational analysis to explore the possibility of using small molecules as binding inhibitors. After some consideration, we chose to first examine crizotinib (Cz). Cz is an ATP mimic that has been shown to interfere with AXL kinase activity, and we wished to know if it had the potential to interact with AXL’s extracellular domain as well. The structure of Cz is displayed in SI Figure S7, and the Vina-generated docking structure, shown in Fig. 3B, has a binding free energy of -7.3 kcal/mol. Careful examination of this image reveals that Cz is located in the same binding pocket as ZENV-DIII which is at the junction between the D1 and D2 domains of AXL-D1D2. Not surprisingly, it has similar hydrophobic interactions as found by ZENV-DIII.

### Roles of Crizotinib and Phosphatidylserine

**Figure 4:**
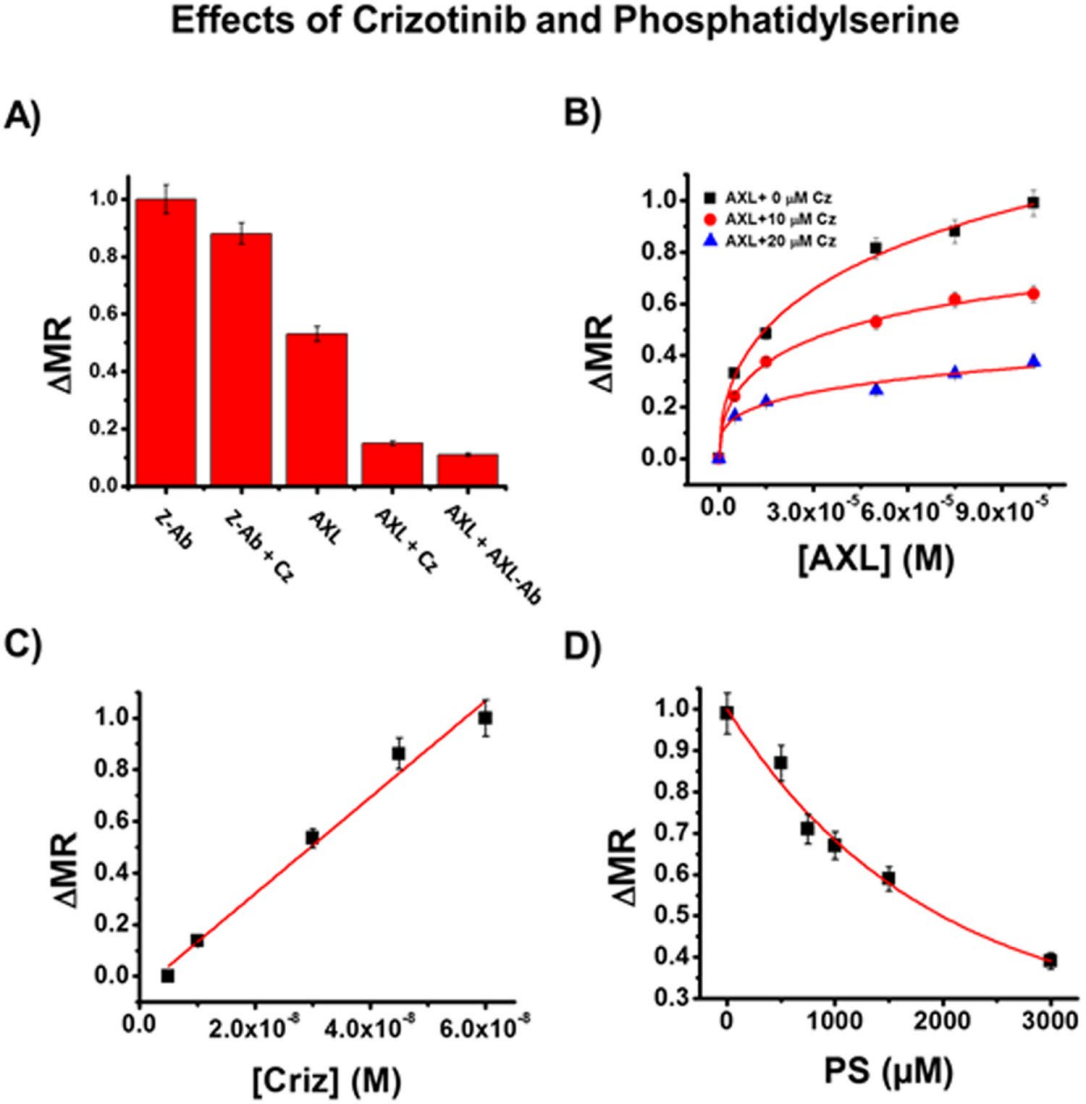
(**A**) ZENV-MRNPs (1.5 mM) were incubated with AXL (3E^−8^ M) alone or in the presence of Cz (1E^−5^ M) or AXL-Ab (1E^−5^ M). As an additional control, ZENV-MRNPs were incubated with Z-Ab alone (3E^−8^ M) and with Z-Ab and Cz (1E^−5^ M), in order to demonstrate Cz’s specificity for AXL. (**B**) ZENV-MRNPs were incubated with AXL (0 to 1E^−4^ M) and Cz (1 to 2E^−5^ µM). (**C**) AXL-MRNPs were incubated with Cz (0 to 6E^−8^ M) for 30 minutes at 25 °C. The positive ∆MR trend reported verifies the interactions between Cz and the extracellular domain of AXL. (**D**) Effects of PS on AXL-ZENV binding. ZENV-MRNPs were incubated with AXL (6E^−5^ M) in the presence of PS (0–3E^−3^ M). Average values of four measurements are depicted ± standard error.

Discovery of functional entry blockers would ideally provide a means to block viral binding, and our sensitive magnetic relaxation platform allows us to screen for molecules with this potential. After computational visualization of these interactions, we conducted corresponding bench-top experiments to study the use of crizotinib (Cz) as a binding inhibitor. We selected an AXL-specific monoclonal IgG antibody (AXL-Ab) and Cz to act as binding blockers while MRnS were conjugated with ZENV. Control solutions were created in which ZENV-MRnS (1.5 mM) were incubated with AXL (3E^−8^ M) in PBS (1X, pH = 7.4) and test solutions were created in which ZENV-MRnS, AXL, and the selected binding inhibitor (10 µM) were incubated together (Fig. 4A). A significant decrease in binding was shown in the solutions containing AXL-Ab and Cz, which is indicative of successful binding inhibition. As an additional control, we tested the effectiveness of Cz to inhibit the binding between ZENV-MRnS and Z-Ab. This showed a relatively minor decrease in binding, suggesting that Cz interacts directly with the extracellular domain of AXL. To explore this interaction further, we conducted a concentration-dependent assay using Cz as an inhibitor. To this end, ZENV-MRnS were incubated with increasing AXL concentrations (0 to 1E^−4^ M) in the presence of Cz (1 to 2E^−5^ M). As seen in Fig. 4B, a higher concentration of Cz reduced the binding between the ZENV-MRnS and AXL more effectively than at lower concentrations. Interestingly, the interaction between Cz and AXL’s extracellular domain has not been shown before. To further demonstrate that Cz is able to directly interact with AXL’s extracellular domain, we performed a binding assay by incubating AXL-MRnS with Cz (0 to 6E^−8^ M) for 30 minutes at 25 °C. The AXL protein used for this experiment did not include the intracellular kinase region, as we wanted to specifically study the interaction between Cz and the extracellular region of AXL. The results (Fig. 4C) indicate a strong binding relationship between Cz and the extracellular region of AXL and will open the door for screening of similar molecules with the potential to serve as entry blockers. It is important to note that the ambiguous pathogenesis of zika makes the effective use of one entry blocker difficult. For instance, recent studies have shown that genetic interference of AXL does not prevent zika entry into neural progenitor cells^33, 34^, and it may be inferred that an AXL inhibitor alone may not be sufficient to fully prevent zika binding and entry. However, these inhibition studies maintain relevancy as they demonstrate the feasibility of this platform to search for additional entry blockers for not only zika, but other pathogens as well.

To further assess the role of phosphatidylserine (PS) mediated apoptotic mimicry and cellular entry of ZIKV, we titrated several-fold excess of PS against the binding experiment between ZENV-MRNPs (1.5 mM) and AXL. Herein, we focused on the potential ability of PS to mediate binding, as seen in other viruses. To this end, ZENV-MRnS were incubated with AXL (60 µM) in the presence of PS (0–3E^−3^ M). The results (Fig. 4D) indicated that ZENV preferentially binds with AXL, and that PS-mediated binding is only pursued when PS is present in high concentrations.

### Effects of temperature and pH on binding

**Figure 5:**
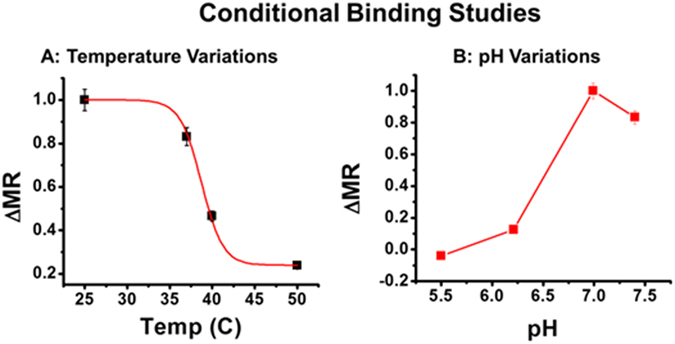
Effects of (**A**) temperature and (**B**) pH on binding between ZENV-MRNPs and AXL. Average values of three measurements are depicted ± standard error.

Following these analytical studies, we examined ZIKV’s ability to remain infective in different temperature (25, 37, 40, and 50 °C) and pH (7.4, 6.99, 6.21, and 5.5) environments. Control solutions containing only ZENV-MRnS (1.5 mM) were used to account for the effects of the temperature or pH on the nanosensors. Our results showed that while binding between AXL and ZENV is strongest at 25 and 37 °C (Fig. 5A), there remained some degree of functionality at 40 °C. The ability to remain infective at higher temperatures is likely due to the reported thermally stable structure of ZENV^22^, which allows for successful binding between ZENV and AXL in harsher temperate conditions. Our pH experiments (Fig. 5B) revealed that while ZENV maintains some functionality at lower pH values, it was strongest at pH = 6.99. The demonstrated ability of ZENV to maintain partial functionality at lower pH suggests that it may also play a secondary role in viral fusion^9^.

## Experimental Section

### Materials and instruments

Ferric chloride hexahydrate, ferrous chloride tetrahydrate, ammonium hydroxide and hydrochloric acid were obtained from Fisher Scientific. DMSO, DMF, N-Hydroxysuccinimide, Polyacrylic acid, and HSP70 were obtained from Sigma-Aldrich. EDC was purchased from Pierce Biotechnology. Dialysis bags (MWCO 6–8 K) were purchased from Spectrum Labs. ZENV peptide, Anti-ZENV antibody, AXL, Anti-AXL antibody, TIM-1, HSP70, and Crizotinib were purchased from Alpha Diagnostics (4adi.com). The magnetic relaxometer (mq20, 0.47 T) was purchased from Bruker and used for T2 data collection. Malvern’s zetasizer-ZS90 was used for the characterization of synthesized IONPs. Magnetic columns were purchased from Miltenyi Biotec and used for purification.

### Synthesis of functional magnetic nanoparticles

#### Synthesis of iron oxide nanoparticles (IONPs)

To synthesize IONPs, three solutions are first created^28^. Solution 1 contains FeCl_3_ (0.622 g), FeCl_2_ (0.334 g), and H_2_O (2 mL). Solution 2 contains NH_4_OH (1.8 mL of 30% stock) and H_2_O (15 mL). The third solution contains polyacrylic acid (0.859 g) and H_2_O (5 mL). Once these solutions are prepared, HCl (90 µL, 12 M) is added to Solution 1, which is then immediately added to Solution 2 while vortexing at 875 RPM. Solution 3 is then added to the mixture and the reaction is continued for 1 hour while vortexing at 3,000 RPM. Following this, the solution is centrifuged for 20 min at 1,620 x g and then 20 min at 2,880 x g to participate the larger particles. Finally, supernatant is dialyzed (molecular weight cutoff: 6–8 K) for purification and the purified IONPs (5 mM) are stored at 4 °C for functionalization. The average diameter of these IONPs were found to 103 ± 2 nm (Figure S8).

#### Conjugation of receptors and antibodies to surface of IONPs

A sample of previously synthesized IONP (5 mL, 5 mM) is diluted with PBS (5 mL, pH = 7.4). Then, EDC (8 mg in 250 µL of MES) is added. Next, NHS (5 mg in 250 µL of MES) is added in small increments within two minutes^28^. Finally, the selected receptor or antibody (10 µL of 1 mM stock) is added. The reaction is then allowed to continue on a table-top mixer for 1 h at room temperature and then overnight at 4 °C. The next day, the solution is purified via magnetic column and the functional MRNPs (1.5 mM) were stored at 4 °C. The conjugation reaction was characterized by measuring the change in size and magnetic relaxation of the functionalized IONPs. For example, the average diameter of ZENV-MRNPs was found to be 110 ± 1 nm (Figure S8) and the increase in magnetic relaxation time (ΔT2) was found to be 18 ms when compared with non-conjugated IONPs. These change in overall diameter and magnetic relaxation further indicated for successful conjugation of ZENV. Conjugation via EDC/NHS chemistry forms amide linkage between the IONP’s surface carboxylic acid groups and primary amine functional groups located on the selected receptor, protein or antibody. In the presence of multiple amine groups, there is a potential for varied conjugate orientation on the surface of the nanoparticle. However, primary amines are favored over secondary and tertiary amines, reducing this variation.

### Assessment of Spin-Spin magnetic relaxation and T2 data normalization

#### Binding Assays

ΔT2 data may be normalized and given the term ΔMR (magnetic relaxation). Normalization to ΔMR values is achieved by dividing the ΔT2 values of samples with various concentrations of the zika envelope protein (ΔT2) by the ΔT2 value associated with the most concentrated solution (ΔT2Max). This normalizes the data to a value of one and the graph will present a linear increase in ΔMR values if there is binding between the nanosensors and the zika envelope. The equation form of this normalization is as follows:

$${\rm{\Delta }}MR=\frac{{\rm{\Delta }}T2}{{\rm{\Delta }}T{2}_{Max}}.$$

#### Competition Assays

Competition assays allow for the determination of apparent dissociation constants (Kd) via the normalization of ∆T2 data to ΔMRComp values. The resulting sigmoidal curve represents the cooperative effect of the competing molecules as they displace the envelope proteins from the nanosensor. ΔMRComp values are determined by subtracting the ΔT2 values obtained with various concentrations of competitor (ΔT2Comp) from the ΔT2 value associated with a solution lacking any competitor (ΔT2Max0), thus scaling the range of values to zero. These values are then divided by the ΔT2Max0, providing a final range of ΔMRComp values that are between 0 and 1. The competitor concentration at which ΔMRComp = 0.5 represents the apparent Kd value. While there are more accurate methods to determine true dissociation constants, the purpose of this assay is to allow for comparative binding analysis between various targeting ligands, rather than the determination of a highly specific Kd value. The final equation used for this normalization is as follows:

$${\rm{\Delta }}M{R}_{Comp}=\frac{{\rm{\Delta }}T{{2}_{Max}}^{0}-{\rm{\Delta }}T{2}_{Comp}}{{\rm{\Delta }}T{{2}_{Max}}^{0}}.$$

### Molecular docking studies

The generation of the AXL structure was accomplished using a BLAST search engine to find structures with similar primary sequence identity with AXL, as previously described. Following this, validation of the structure and all-atom contact analysis were subsequently conducted using Molprobity (Legacy version 4.02)^35^. Molprobity analyzes all-atom contacts for clashes, and bond angles and bond lengths for deviation from the norm. Amino acids that contain such deviations are listed as outliers. Structures validated by Molprobity are given an overall Ramachandran score from 1 to 100%, with scores above 90% are normally associated with acceptable structures. Further validation of the AXL-D1D2 structure was obtained using the Yasara plugin of the “Multiple Structure alignment algorithm”, MUSTANG to align the AXL-D1D2 modelled structure to the Tyro3 crystal structure^31^. MUSTANG is a structural alignment algorithm, which performs sequence alignment as well as structural superposition based on special arrangements of the C atoms. Docking analysis was initially performed using GRAMM-X protein-protein docking and Rosetta online servers, and Autodock Vina. Further details regarding the computational analysis can be found in the supplemental information.

## Conclusions

In summary, we have introduced a novel application of magnetic relaxation technology that allows for the rapid and sensitive analysis of simple host-pathogen interactions. In our studies, we first demonstrated the effectiveness of this platform using a well understood antibody-antigen interaction, and later examined a number of receptors for activity with ZENV. We have verified that ZENV is able to bind with both HSP70 and TIM-1 in addition to AXL. Although it appears that ZENV favors AXL as a binding candidate, we believe that it is crucial to heed its potential for multi-avenue binding, as it may play a vital role in the appearance of zika’s array of symptoms. The computational analysis of molecular docking between ZENV and AXL established their possible binding sites and calculated binding energy for the first time. These experiments helped finding crizotinib as a possible small molecule inhibitor, which was then verified using our nanoplatform. Additionally, we have explored the possibility of using AXL-antibodies or Cz as entry inhibitors, and have demonstrated that PS may have the potential to mediate viral entry when present in high concentrations. Furthermore, we have further characterized these binding behaviors, providing a range of temperature and pH parameters that affect binding. Further studies are currently underway to screen for additional receptor candidates and potential therapeutics, as well as to study the role of GAS6 in AXL activity. Our easy-to-use magnetic relaxation platform may be applied to any number of pathogen systems in the future and will allow us to learn more about such intricate molecular interactions. This will be crucial as we continue to seek more in-depth answers regarding pathogenesis and inhibition of not only zika, but other viral and bacterial pathogens as well.

## Electronic supplementary material

Supporting Information
