# Supplementary material for: Highly Sensitive, Engineered Magnetic Nanosensors to Investigate the Ambiguous Activity of Zika Virus and Binding Receptors
Source: Sci Rep. 2017 Aug 7;7:7377. doi: 10.1038/s41598-017-07620-y (PMC5547150; doi:10.1038/s41598-017-07620-y)
Supplement: Supplementary file 1 — Supporting Information [file 41598_2017_7620_MOESM1_ESM.pdf]

Supporting Information

Highly Sensitive, Engineered Magnetic Nanosensors to Investigate the Ambiguous Activity of Zika Virus and Binding Receptors

*Tyler Shelby,‡ Tuhina Banerjee,‡ Irene Zegar and Santimukul Santra**

Department of Chemistry, Pittsburg State University, 1701 S. Broadway Street, Pittsburg, KS 66762, USA

*Corresponding author: Santimukul Santra. *E-mail: ssantra@pittstate.edu*

Magnetic relaxation data.

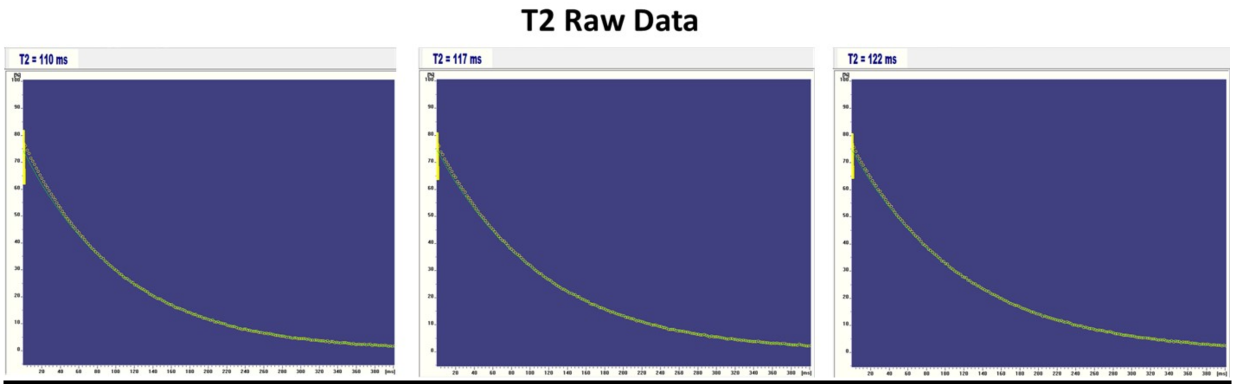

Figure S1: Raw T2 data obtained by incubating Z-Ab conjugated nanosensors with ZENV (0, $1E^{-10}$, and $1E^{-9}$ M, respectively).

Interaction between zika-Ab and hemagglutinin protein:

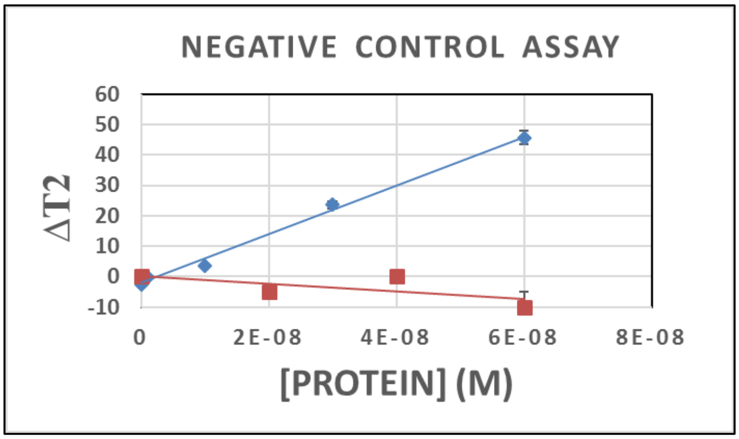

Figure S2: For this experiment, Z-Ab-IONPs (1.5 mM) were incubated with increasing concentrations of Hemagglutinin, which was selected for non-reactivity with Z-Ab. This binding is represented above by the negative orange line, and is compared to a positive binding trendline obtained by incubating Z-Ab-IONPs with increasing concentrations of ZENV (0 to 6E^{-8} M). This data verifies that our nanosensors only bind to proteins in solution that have binding affinities for proteins that have been conjugated to the surface of our IONPs. Average values of three measurements are depicted ± standard error.

Binding assays in plasma:

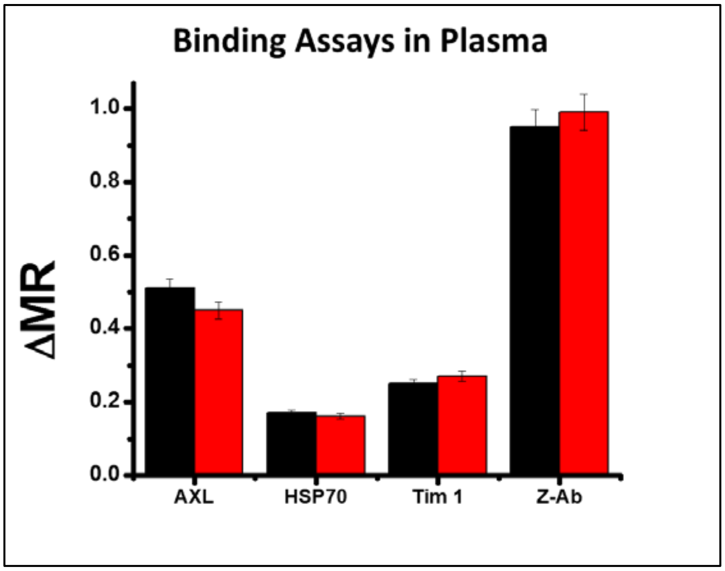

Figure S3: Binding assays with receptor-bound and Z-Ab-bound IONPs in solution with PBS (black) and Plasma (red) for comparison. Binding is shown to behave similarly in both environments. Average values of three measurements are depicted ± standard error.

Receptor Competition Assays:

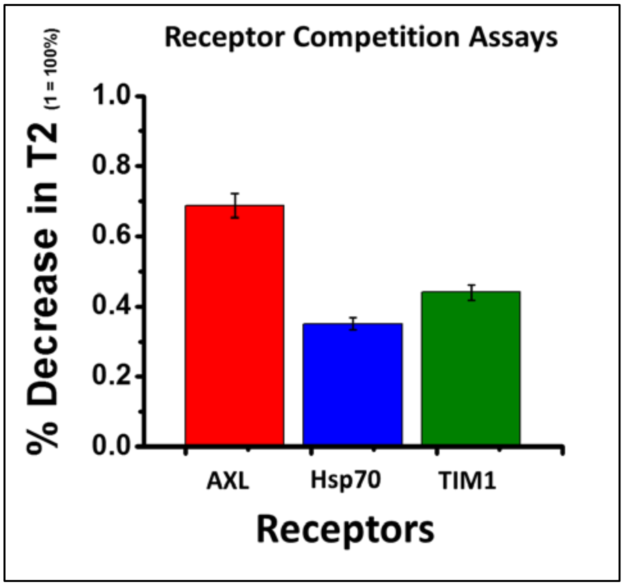

Figure S4: Competition assays were conducted via the addition of non-bound target receptors to the respective test solutions, resulting in decreased T2 values. To this end, receptor-bound nanosensors (1.5 mM) were incubated in PBS (1X, pH = 7.4) with ZENV ($3E^{-8}$ M) and the corresponding free receptor ($1.8E^{-7}$ M). For example, AXL-IONPs were incubated with ZENV and free AXL. These free-floating receptors are able to bind to the envelope proteins in solution, disrupting their interactions with the nanosensors. This verifies that the binding seen is due to interactions between ZENV and receptor, rather than the coating of the nanosensor. The corresponding decrease in T2 values can then be used to comparatively analyze the binding affinities between ZENV and the selected cell receptors. The greater the decrease in T2, the stronger the interaction between the free receptor and ZENV.[5] As shown, the AXL solution showed a greater decrease in T2, followed by TIM1, and finally HSP70. This information verifies the trend reported in **Figure 2C**, and once again demonstrates ZENV's preference for AXL. Average values of three measurements are depicted ± standard error.

Computational analysis:

To model the interaction between AXL-D1D2 and Domain III of the Zika envelope protein (ZENV-DIII), the coordinates for ZENV-DIII (**Figure S5**) were taken from the coordinate file of the crystal structure for ZENV- DIII bound to the Zika specific antibody ZV-48 (PDB ID: 5KVE). Molecular docking of ZENV-DIII to AXL-D1D2 (**Figure S6**) was conducted, first using the GRAMM-X Protein-Protein Docking Web Server v.1.2.0 to obtain a reasonable guess of the binding site[1]. The docked structure with the most stable energy was then submitted to the Rosetta online server (http://rosie.rosettacommons.org)[2]. Rosetta docking was performed using the protein-protein Docking2 protocol which determines the structures of protein-protein complexes by using rigid body perturbations. The Rosetta output file contained 1000 predicted models along with overall energies for each model and interaction energies between ZENV-DIII and AXL-D1D2. The model with the lowest overall energy and the lowest interaction energy is subjected to energy minimization using the FoldX-RepairPDB routine.

The 3D structure of crizotinib (**Figure S7**) used to model its interaction with AXL-D1D2 was obtained from the PubChem Compound Database[3]. Molecular Docking of crizotinib to ALX-D1D2 was performed using the Autodock Vina plugin in the PyRx Virtual Screening Tool Version 0.9.4[4]. The gridbox used to define the binding site of ALX-D1D2 was chosen to encompass the entire protein structure in order not to bias the docking. The model with the lowest binding energy ($\Delta G_{binding}$) was chosen to represent the structure with the strongest interaction of crizotinib to AXL-D1D2. This structure was energy minimized using the RepairPDB in the FoldX plugin in Yasara. The structure figures used in this manuscript were made using Yasara View or AutoDockTools-1.5.6[5].

The all-atom analysis of the AXL-D1D2 structure determined using modeller (**Figure S6**) gave a Ramachandran favored score of 94.6% which indicates that there are no major outliers in this structure that significantly affect its validity. Furthermore, the generated structure of AXL-D1D2 was compared with the crystal structure of Tyro3 using MUSTANG, which gave a 1.11 RMSD value, indicating a high structural similarity between the two proteins. The AXL-D1D2 structure was subsequently used in Rosetta docking as a receptor for ZENV-DIII. The resulting ZENV-DIII/AXL-D1D2 docked structure (**Figure 3A**) was analyzed using Molprobity, which gave a Ramachandran score of 94.1%, indicating high structural favorability. Furthermore, the interaction energy

between ZENV-DIII and AXL-D1D2 determined to be -8.8 kcal/mol, which corresponds to a K_d value of $3.1E^{-7}$ M^{-1}. Careful examination of this structure indicates that ZENV-DIII lies in a pocket formed by a proline-rich D2 region near the junction between the D1 and D2 domains of the AXL-D1D2 dimer. Two hydrophobic interactions were detected between ZENV-DIII and AXL-D1D2 homodimer. These interactions are between Pro137 of the AXl-D1D2 homodimer and ASP333 of ZENV-DIII, and Pro154 of the AXL-D1D2 homodimer and Val358 of ZENV-DIII. It is interesting to note that D2 of the Ig-like domain region of AXL is proline rich with a high degree of conservation in this region among the AXL/Tyro3 family of receptor tyrosine kinases. The two prolines involved in hydrophobic interactions with ZENV-DIII are part of a cluster of prolines that are found to adopt a cis-conformation and have been implicated as being part of the binding site of GAS6[6].

The structure representing the binding of crizotinib to AXL-D1D2 is shown in **Figure 3B**. The binding energy of crizotinib to AXL-DIDII was determined to be -7.3 kcal/mol, which is comparable to the interaction energy obtained for the ZENV-DIII and AXL-D1D2. Furthermore, careful examination of **Figure 3B** shows that crizotinib is located in the same binding pocket as ZENV-DIII which is at the junction between the D1 and D2 domains of AXL-D1D2 and it has similar hydrophobic interactions as found by ZENV-DIII. One particular interaction is between crizotinib and Pro154, one of the proline residues that are highly conserved among the AXL/Tyro3 family. It is interesting to note that the other nine vina generated structures with comparable binding constants are also found to bind in various positions in the same pocket of ZENV-DIII.

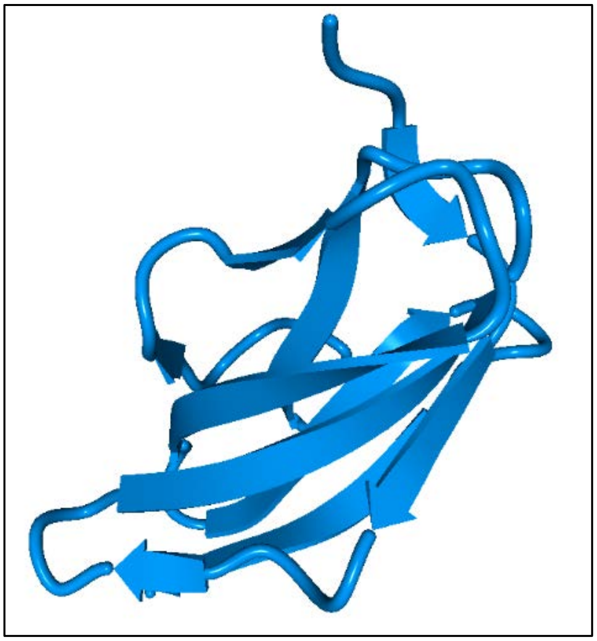

Figure S5: The structure of ZENV-DIII adapted from the crystal structure for ZENV- DIII bound to the Zika specific antibody ZV-48 (PDB ID: 5KVE).

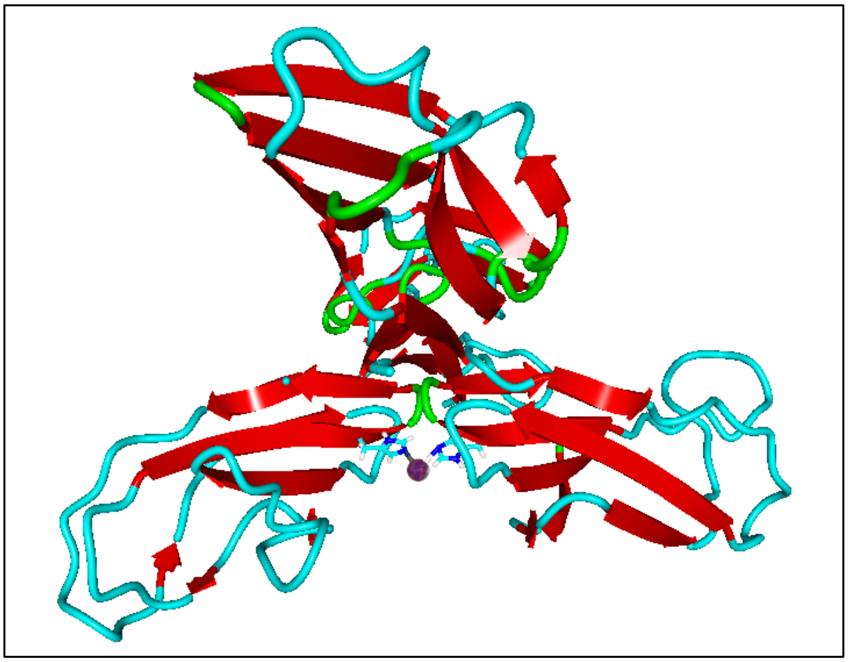

Figure S6: The AXL-D1D2 structure obtained using Modeller 9.17.

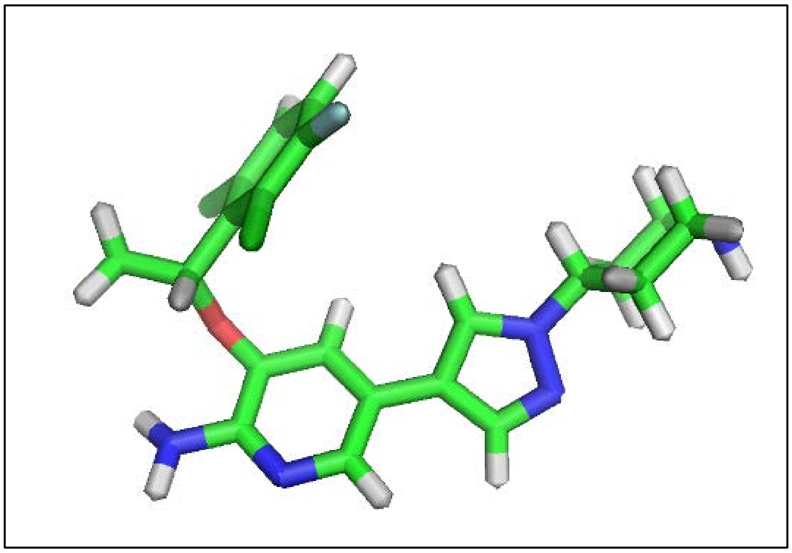

Figure S7: The 3D structure of crizotinib used in the AXL-D1D2 docking study

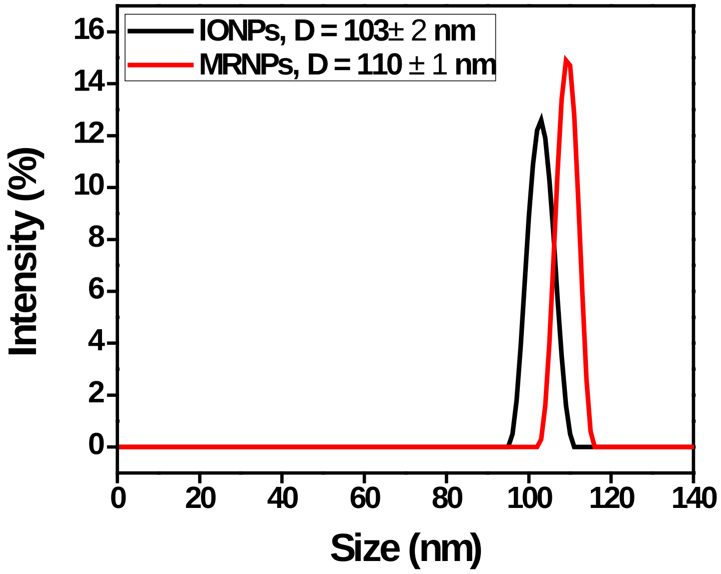

Figure S8: Measurement of size of IONPs (Diameter D = 103 ± 2 nm) and ZENV functional MRNPs (D = 110 ± 1) using dynamic light scattering. Average values of four measurements are depicted ± standard error.

References:

1. Tovchigrechko, A. & Vakser, AI. GRAMM-X public web server for protein–protein docking. *Nucleic Acids Res.* **34,** W310 (2006).

2. Trott, O. & Olson A. AutoDock Vina: improving the speed and accuracy of docking with a new scoring function, efficient optimization, and multithreading. *J. Comp. Chem.* **31,** 455 (2010).

3. National Center for Biotechnology Information. PubChem Compound Database; CID=11626560, https://pubchem.ncbi.nlm.nih.gov/compound/11626560 (accessed Dec 19, 2016).

4. Krieger, E. & Vriend, G. YASARA View - molecular graphics for all devices - from smartphones to workstations. *Bioinformatics* **30,** 2981 (2014).

5. Sanner, M. F. Python: A Programming Language for Software Integration and Development. *J. Mol. Graphics Mod.* **17,** 57 (1999).

6. Heiring, C., Dahlback, B. & Muller, Y. A. Ligand recognition and homophilic interactions in Tyro3: structural insights into the AXL/Tyro3 receptor tyrosine kinase family. *J. Biol. Chem.* **279,** 6952 (2004).
